# microRNAs identified in prostate cancer: Correlative studies on response to ionizing radiation

**DOI:** 10.1186/s12943-020-01186-6

**Published:** 2020-03-23

**Authors:** Maureen Labbé, Christianne Hoey, Jessica Ray, Vincent Potiron, Stéphane Supiot, Stanley K. Liu, Delphine Fradin

**Affiliations:** 1 CRCINA, INSERM, Université d’Angers, Université de Nantes, Nantes, France; 2 Department of Medical Biophysics, University of Toronto, Toronto, Ontario Canada; 3 Biological Sciences, Sunnybrook Research Institute, Sunnybrook Health Sciences Centre, Toronto, Ontario Canada; 4 Institut de Cancérologie de L’Ouest René Gauducheau, Saint-Herblain, France; 5 Department of Radiation Oncology, University of Toronto and Odette Cancer Centre, Sunnybrook Health Sciences Centre, Toronto, Ontario Canada

**Keywords:** microRNA, prostate cancer, radiotherapy, radiation resistance, biomarkers

## Abstract

As the most frequently diagnosed non-skin cancer in men and a leading cause of cancer-related death, understanding the molecular mechanisms that drive treatment resistance in prostate cancer poses a significant clinical need. Radiotherapy is one of the most widely used treatments for prostate cancer, along with surgery, hormone therapy, and chemotherapy. However, inherent radioresistance of tumor cells can reduce local control and ultimately lead to poor patient outcomes, such as recurrence, metastasis and death. The underlying mechanisms of radioresistance have not been fully elucidated, but it has been suggested that miRNAs play a critical role. miRNAs are small non-coding RNAs that regulate gene expression in every signaling pathway of the cell, with one miRNA often having multiple targets. By fine-tuning gene expression, miRNAs are important players in modulating DNA damage response, cell death, tumor aggression and the tumor microenvironment, and can ultimately affect a tumor’s response to radiotherapy. Furthermore, much interest has focused on miRNAs found in biofluids and their potential utility in various clinical applications. In this review, we summarize the current knowledge on miRNA deregulation after irradiation and the associated functional outcomes, with a focus on prostate cancer. In addition, we discuss the utility of circulating miRNAs as non-invasive biomarkers to diagnose, predict response to treatment, and prognosticate patient outcomes.

## Introduction

Prostate cancer (PCa) is the second most common cancer and the fifth leading cause of cancer death in men worldwide, with an estimated 1.3 million new cases and 359,000 deaths per year [1]. Currently, treatment options for localized disease are active surveillance, prostatectomy, or radiotherapy, with or without hormone therapy [2, 3]. Despite curative radiation regimens, radioresistance and clinical relapse is reported in numerous cancer types [4–7], including PCa [8]. Understanding the molecular events that cause radioresistance in PCa can lead to the development of improved therapies.

Ionizing radiation (IR) induces biological effects in both tumor cells and the surrounding tumor microenvironment (TME) (Fig. . 1). On a cellular level, IR produces DNA damage, both directly from ionization and indirectly by generation of Reactive Oxygen Species (ROS) [9]. DNA damage triggers the DNA Damage Response (DDR) to repair damaged DNA, or induces cell cycle arrest and cell death if repair is not possible [10, 11]. As cell cycle checkpoints are frequently dysregulated in cancer, radiotherapy exploits this vulnerability to produce greater unrepaired DNA damage and subsequent cell death within tumor cells. The biological impact of IR is greatly affected by hypoxia within the target site, which influences the amount of DNA damage caused by IR-induced ROS. Hypoxia is a pathological state where the tissue has low levels of oxygen reaching the cells. Under hypoxic conditions, hypoxia-inducible factor 1α (HIF-1α) is stabilized and allows for the transcription of genes involves in tumor survival and progression. Chronic hypoxia has also been demonstrated to impair DNA repair through downregulation of the repair machinery resulting in genomic instability which may select for a more aggressive cancer phenotype [12]. To partly counter the negative impact of a hypoxic TME on IR, a fractionated radiotherapy course is typically employed (i.e., treatments delivered daily over several weeks). This fractionated treatment eliminates the well oxygenated tumor cells, which then allows for hypoxic cells to reoxygenate and become radiosensitive [13–15]. After all, oxygen is the best radiosensitizer due to its role in generating ROS. IR also affects the TME [16], notably by modifying the vasculature large doses of radiation may promote endothelial cell apoptosis [17], and vascular collapse, whereas smaller doses of fractionated IR can promote vascular maturation and improved perfusion [18]. Tumor response to IR can be modulated by other cells through two phenomenon: the bystander effect, where non-irradiated cells are negatively impacted by adjacent irradiated cells; and the abscopal effect where the immune system becomes primed to eradicate tumor cells at sites distant from the irradiated site [19]. Indeed, IR can lead to immunogenic cell death, as immune cells utilize antigens generated from damaged tumor cells to activate dendritic cells (DCs) and CD8+ cytotoxic T cells to kill tumor cells [20]. Interestingly, hypoxia promotes transcription of the androgen receptor (AR) expression [21], which is a key moderator of PCa proliferation and survival [22].

**Fig. 1:**
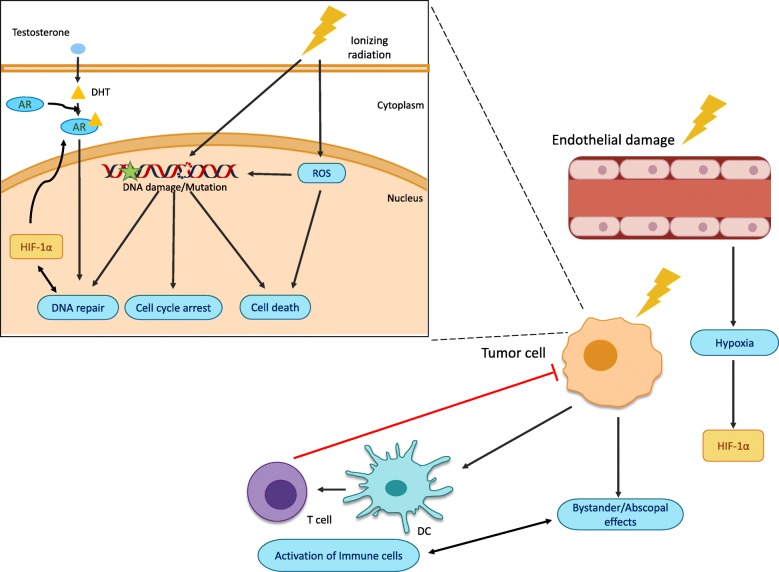
Radiation effects on tumor cells and the tumor microenvironment. Damage induced by ionizing radiation lead to numerous cell effects in the cell and within the tumor microenvironment (TME). Irradiation causes DNA damage which triggers DNA damage response to repair the damaged DNA, induce cell cycle arrest or cell death. Reactive oxygen species (ROS), produced following irradiation, are also implicated in radiation responses. In the TME, tumor endothelial cells sensitive to irradiation undergo apoptosis resulting in vascular destruction and hypoxia. Hypoxia stimulates DNA repair through the androgen receptor (AR) leading to less faithful DNA repair and accumulation of mutations. Radiation also promotes bystander and abscopal effects. One of these changes is an increase of tumor cell antigen availability which activates dendritic cells (DC) and T cells to eradicate tumor cells. DHT, dihydrotestosterone; HIF-1α, hypoxia-inducible factor-1α

Due to this central role of AR in proliferation, survival, and treatment response [23, 24], Androgen Deprivation Therapy (ADT) is commonly used in synergy with RT [25] as a therapy for PCa due to its vital role in PCa progression. Despite the initial response to ADT, most patients develop resistance and progress to a more aggressive form of PCa, referred as Castration-Resistant Prostate Cancer (CRPC). Interestingly, CRPC cells upregulate the expression of AR (notably AR spliced variants leading to constitutively active AR) and overtime acquire radioresistance. AR signaling enhances DNA DSB repair through the non-homologous end-joining (NHEJ) pathway [26], and induces the over-expression of ataxia-telangiectasia mutated (ATM) in CRPC cells [27]. In CRPC cells, including the radioresistant ones, activated CDC42 kinase 1 (Ack1) is over-expressed. Ack1 is a kinase implicated in the phosphorylation of pTyr^267^-AR, which is critical to androgen-independent AR transactivation and tumor promoting. During this process, pTyr^267^-AR is recruited to the ATM enhancer to up-regulate ATM, which could lead to radioresistance. It has also been shown that IR can induce expression of AR in a dose-dependent manner, through modulating nuclear translocation and increasing transcriptional activity [28], which may promote radioresistance.

Research into the molecular mechanisms governing radioresistance continues to shed new light onto this important clinical problem. A class of non-coding RNAs (ncRNAs) termed microRNAs (miRNAs) are believed to play a key role in the regulation of radiation response, and their expression has been linked to radioresistance in many cancers [29–33]. Thus, further investigation into the radio-modulating role of miRNAs in PCa is important. Indeed, altered miRNA expression occurs rapidly after IR [34, 35] and leads to rapid changes in protein levels of the targeted mRNAs. miRNAs are ncRNAs of approximately 19 to 22 nucleotides in length that negatively regulate gene expression at the post-transcriptional level [36]. Association of a miRNA with the protein Argonaute (AGO) forms an RNA-induced silencing complex (RISC), which then binds the mRNA target primarily on the 3’untranslated region (3’UTR) [37], and decreases gene expression via translational repression, mRNA cleavage, or destabilization of the target mRNA [38]. miRNAs have been shown to modulate virtually all cellular processes including cell cycle control, proliferation, and differentiation [39], and can consequently impact the response to radiotherapy through influencing these pathways. Nevertheless, the number of studies on the regulatory mechanisms of miRNA in PCa radioresistance is quite limited, and our understanding of the functional role exerted by these miRNAs is only beginning to be elucidated. Furthermore, there is a significant clinical need to find non-invasive biomarkers to improve the management of PCa, and circulating miRNAs may serve to fulfill this need.

In this review, we will first provide an up-to-date summary of the literature on miRNAs that are influenced by radiotherapy in PCa. We will then examine the role of miRNAs in cellular response to radiation, and discuss the utility of using circulating miRNAs as non-invasive biomarkers.

### miRNAs in response to radiotherapy

It is known that miRNA expression can be modulated by IR. This can occur in PCa cells as well as surrounding cells exposed to radiation treatment [31]. Numerous studies, using Next Generation Sequencing (NGS) or microarray technology, have investigated miRNA expression in cells before and following exposure to IR (Table 1). We observed large differences between studies, including the radiation dose, the technology and methodology used, and perhaps most notably the miRNA expression patterns in cells before IR. Due to these variations, the same miRNA is often identified in the literature as being both up- and down-regulated after irradiation, depending on the study. For example, miR-141 has been shown to be up-regulated in LNCaP cells [49] and down-regulated in 22RV1-radioresistant (RR) cells [50]. Interestingly, circulating miR-141 is sometimes up-regulated in PCa patients before treatment when compared with healthy controls [56–58]. A high expression level of miR-141 in human PCa surgical specimens (n=535) is associated with reduced biochemical or clinical failure-free survival [59]. In human PCa cell lines where miR-141 is under-expressed, Liu *et al.* have recently identified its target by whole-genome RNA sequencing (RNA-seq) such as multiple pro-metastasis genes like *CD44, Rho GTPase* and enhancer of zeste 2 polycomb repressive complex 2 subunit *(EZH2*) [60]. These results highlight the contradiction action of miR-141 in PCa and suggest that one miRNA could act as oncomiR or suppressor of tumor in PCa.

**Table 1:** miRNAs dysregulated by irradiation in prostate cancer cells

miRNA after irradiation						Functional role
Expression	miRNAs	Cell lines	Doses	Methods	References	
*Increased*	let-7e, miR-18b miR-92a-1, miR-92a-2, miR-320a, miR-365-1, miR-365-2	PC-3-RR cells	2 Gy x 45, fractionnated	NGS	[40]	-
	miR-95					Radioresistance [40]
	miR-9, miR-22, miR-25, miR-550a, miR-548h	PC-3 cells	10 Gy	NGS	[41]	-
	miR-30a					Radiosensitization [42]
	let-7 family, miR-34a, miR-146a	PC-3 and LNCaP cells	0.5 Gy x10, 1 Gy x10, fractionned	microarray	[43]	-
	miR-16	LNCaP cells	0.5 or 4 Gy	microarray	[44]	Radiosenzitization [45]
	miR-34c, miR-372, miR-520c, miR-520f	LNCaP cells	6 Gy	microarray	[46]	-
	miR-449					Radiosensitization [47, 48]
	miR-9-1, miR-22, miR-24, miR-29b, miR-141, miR-191, miR-200c	LNCaP cells	6 Gy	microarray	[49]	-
	miR-30a					Radiosensitization [42]
	51 miRNAs increased notably miR-29a, miR-130a, miR-4521	22RV1-RR compared to 22RV1 cells	60 Gy, (2 Gy fractioned doses)	microarray	[50]	-
	miR-221, miR-222					Radioresistance [51]
	miR-34c, miR-154*, miR-379, miR-383, miR-488	C4-2 cells	6 Gy	microarray	[46]	-
*Decreased*	let-7c, let-7d, let-7e , miR-15a, miR-30d, miR-92a, miR-125a, miR-197, miR-221, miR-320b, miR-342, miR-361, miR-374a, miR-501, miR-671	PC-3 cells	10 Gy	NGS	[41]	
	miR-17					Radiosensitization [52]
	miR-17-92 cluster	PC-3, LNCaP and DU145 cells	5 and 10 Gy, single dose and 0.5 Gy x 10, 1 Gy x 10 fractionned dose	microarray	[43]	-
	miR-100	LNCaP cells	6 Gy	microarray	[46]	Radiosensitization [53]
	miR-107, miR-122a, miR-133b, miR-187, miR-196a, miR-487					-
	miR-145					Radiosensitization [54]
	miR-521					Radiosensitization [55]
	miR-106b	LNCaP cells	6 Gy	microarray	[49]	Radioresistance [55]
	miR-199a					-
	miR-133b, miR-135b, miR-143, miR-196a, miR-218, miR-521	C4-2 cells	6 Gy	microarray	[46]	-
	46 miRNAs decreased notably miR-141, miR-3607, miR-4284	22RV1-RR compared to 22RV1 cells	60 Gy (2 Gy fractioned doses)	microarray	[50]	-

*RR* radioresistant, *NGS* Next-Generation Sequencing

Additional prominent IR-responsive miRNAs are members of the let-7 family, whose expression is frequently found to be altered by IR, however, this is not surprising since the mature members of this family are the most abundant among all miRNAs in the cell [40, 41, 43]. The let-7 family is most commonly described as a tumor suppressor family as they inhibit the expression of multiple oncogenes such as *KRAS* [61] and *Myc* [62].

Interestingly, PCa miRome could also be modulated by AR. Indeed, the AR by binding to androgen response elements (AREs) can directly regulate miRNA expression [63]. miR-21, known to induce radioresistance [64] and to play a role in CRPC [65], is a miRNA regulated by the AR [66].

Definitively concluding a miRNA is up- or down-regulated by IR is difficult since findings are heavily influenced by the variations in methodology between research groups. As technology such as NGS becomes more accessible, larger datasets will hopefully help to decipher complex changes of miRNA expression following radiation and identify potential patterns which can be utilized clinically to evaluate radiation response.

### miRNAs in DNA repair mechanisms induced by radiotherapy

IR induces DNA damage including double-strand breaks (DSBs), the most deleterious to cell survival. A major mechanism of radioresistance in cancer cells is altered expression of DDR components and DNA repair pathway such as NHEJ or homologous recombination (HR). Numerous studies have shown that miRNA expression changes in response to DNA damage in order to regulate DDR and DNA repair pathways [29, 33, 67].

**Fig. 2:**
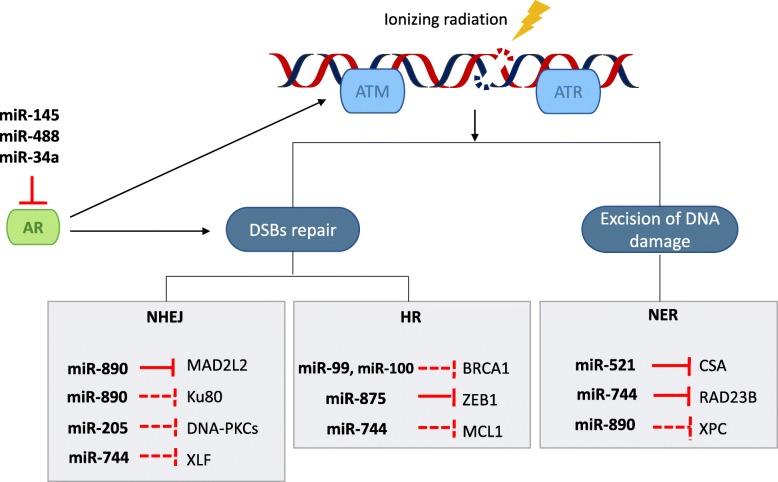
Modulation of DNA Damage by miRNAs in response to irradiation in prostate cancer. Radiation induces DNA damage. In order to repair DNA damage, the cell initiates DNA damage response (DDR) pathways. miRNAs, whose expression are modulated by irradiation, are key players in increasing or inhibiting DDR in PCa radiation response by targeting the mediators, transducers or effectors of DDR. ATM, ataxia-telangiectasia mutated; ATR, ataxia telangiectasia and Rad-3-related protein; DSB, double-strand breaks; AR, androgen receptor; NHEJ, Non-Homologous End Joining; HR, Homologous Recombination; NER, Nucleotide Excision Repair. Inhibition line indicates direct targeting and dashed-inhibition line indicates indirect targeting

To identify the impact of miRNAs on DNA repair and radioresistance, Hatano *et al.* transfected 810 different miRNA mimics separately into LNCaP-MLuc cells and then irradiated the miR-transfected cells with 4 Gy dose [55]. Eleven days after radiation treatment, MLuc activity was measured to determine cell viability. Among the miRNAs studied, 75 were categorized as radioprotective, in particular the miR-106b family, while 324 miRNAs were identified as radiosensitizing, notably miR-521. Further investigations on the candidate miRNAs highlighted in this screen need to be performed to verify and characterize their influence on DDR and DNA repair. For example, the role of miR-521 in radiosensitivity of PCa cells (C4-2 and LNCaP) was previously described by Josson *et al.*, as it was down-regulated after radiation treatment, and further experiments identified a DNA repair protein, cockayne syndrome protein A (CSA), as a potential target of miR-521 [46]. Several other miRNAs have been shown to impair DNA repair through targeting repair response proteins. miR-890 and miR-744-3p directly target the DNA repair proteins mitotic arrest deficient 2 like 2 (MAD2L2) and RAD23 homolog B (RAD23B) respectively, in addition to indirectly reducing additional DDR proteins such as Ku80, xeroderma pigmentosum complementation group C (XPC), XRCC4-like factor (XLF) and cell leukemia 1 (MCL1) [55] (Fig. 2). *In vivo*, miR-890 mimic slows down the growth of PCa xenografts following IR treatment when compared with miRNA control and leads to a radiosensitive phenotype [55]. More recently, El Bezawy *et al.* showed that the over-expression of miR-205 in DU145 and PC-3 cell lines induced an increased sensitivity to radiation by impairing the ability of these cell lines to repair post-IR DNA damage, and identified Protein Kinase C epsilon (PKCε) as a direct target of this miRNA [68]. PKCε is known to trigger nuclear Epidermal Growth Factor Receptor (EGFR) accumulation, leading to the activation of DNA-dependent protein kinase (DNA-PK) [69].

Regarding miRNAs altering homologous recombination, Mueller *et al*. showed that miR-99a and miR-100 are down-regulated in radioresistant PCa cells and up-regulated following IR-induced DNA damage. The overexpression of these two miRNAs reduced the efficiency of DSB repair and increased *in vitro* radiosensitivity by targeting SNF2H (SWI/SNF-related matrix-associated actin-dependent regulator of chromatin subfamily A member 5, or SMARCA5), a chromatin-remodeling factor which recruits Breast cancer susceptibility gene 1 (BRCA1) to sites of DSBs [53]. Another tumor suppressor miRNA targeting DDR is miR-875-3p, which induces radiation sensitivity in PCa cells by inhibiting HR by regulating checkpoint kinase 1 (CHK1) expression and through down-regulation of Zinc Finger E-box-binding homeobox 1 (ZEB1), a protein implicated in epithelial to mesenchymal transition (EMT) [70] (Fig. 2).

It is also interesting to note, that the AR interacts with, affects, and is affected by DDR proteins, resulting in increased, but less faithful, repair of DNA damage and therefore formation of mutations [26, 71]. miR-145 directly targets *AR* [72] and was shown to be down-regulated after IR in LNCaP cells [46], while miR-488 and miR-34a have also been found to target the AR mRNA [43, 46, 73–75], suggesting they could play a role in radiation sensitivity through regulating AR-DDR feedback loops (Fig. 2).

### miRNAs in cell cycle progression after radiation

Following IR-induced DNA damage, cell cycle progression is arrested at G1 and G2 checkpoints to allow time for the cell to repair this damage. Typically once DNA is repaired, the cell will re-enter the cell cycle, however, if it is unable to repair the damage it will undergo cell death (discussed in more detail in the following section) [76]. Cell cycle progression past checkpoints depends on cyclins, cyclin dependent kinases (CDKs), inhibitors, and also on transcription factors such as the E2F family, and each of these components can be regulated by miRNAs.

**Fig. 3:**
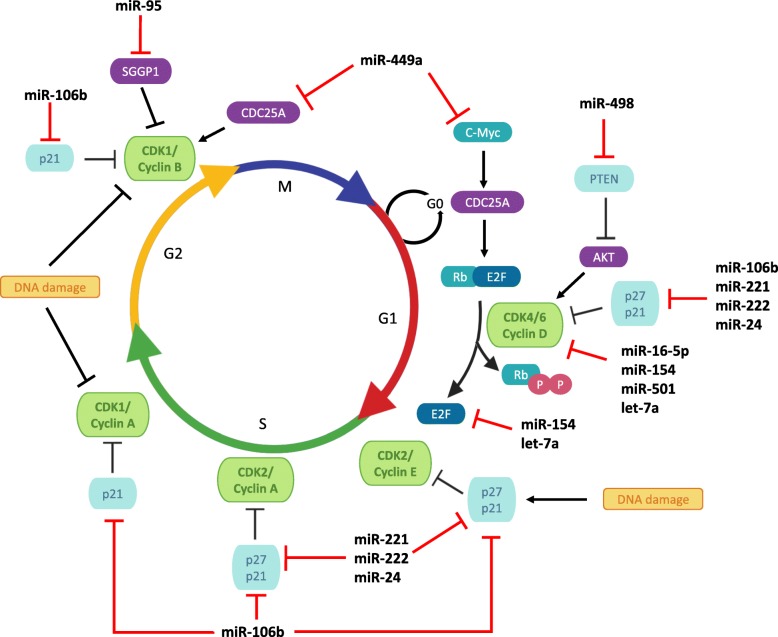
Regulation of cell cycle progression by miRNAs in prostate cancer radiation response. Following DNA damage induced by irradiation, cell cycle arrest is initiated in order to allow DNA damage repair. Cell cycle progression depends on cyclin dependent kinases (CDKs), cyclins, inhibitors and also transcription factors family E2F, themselves regulated by miRNAs, whose expression could be modulated by irradiation. S, S-phase; M, Mitosis; G1 and G2 indicate transition phases of the cell cycle; G0 indicates quiescent cells; PTEN, Phosphatase and TENsin homolog; CDC25A, Cell division cycle 25 A; Rb, Retinoblastoma protein; AKT, protein kinase B; P, phosphorylation; SGGP1, sphingosine-1-phosphate phosphatase 1

It has been suggested that one of the cyclins, cyclin D, is regulated after IR by several miRNAs, which are all overexpressed in response to IR. Cyclin D is involved in Retinoblastoma (Rb) protein phosphorylation to promote cell cycle progression [44]. Wang *et al*. demonstrated that miR-16-5p induces cell cycle arrest at G0/G1 phase by targeting cyclin D1 in irradiated PCa cells [44], which was later confirmed by Takeshita *et al*. in mouse bone tissues [77] (Fig. 3). Cyclin D1 is also indirectly suppressed by cell cycle-related and expression-elevated protein in tumor (CREPT) which is targeted by miR-501 [41, 78]. The IR-induced down-regulation of miR-501 might further prevent cell cycle progression, and thereby radiosensitize PCa cells to radiotherapy. Two others miRNAs, let-7a and miR-154, are known to target cyclin D, isoform 2 [43, 46, 79–81]. As such, these miRNAs may act as radiosensitizers in PCa cells and might induce cell cycle arrest via cyclin D.

In PCa, the G2/M checkpoint can also be dysregulated directly by miRNAs following IR, for example miR-95 overexpression may enhance transit through the G2/M phase by targeting Sphingosine-1- phosphate phosphatase 1 (SGPP1 ) [40], which contributes to radioresistance. In contrast, miR-449a has been shown to induce cell cycle arrest at G2/M phase and enhance radiosensitivity *in vitro* and *in vivo* [47] (Fig. 3). Using an antagomir (anti-miR-449a), miR-499a expression was suppressed and increased cell proliferation was observed [48]. Conversely, after over-expressing miR-449a in PC-3 and DU145 cells using a plasmid construct, cell cycle arrest was observed [47]. Collectively, these results support the hypothesis that miR-499a is involved in cell cycle arrest, which impacts radiosensitivity. Further investigations identified Cell division cycle 25A (Cdc25A), a protein implicated in cyclin B activation and a mediator of *c-Myc* function, as a direct target of miR-449a. *c-Myc,* which controls Cdc25A expression, is also observed as a target of miR-449a. Surprisingly, in the first publication by Mao *et al.*, miR-449a is decreased in PCa DU145 and PC-3 cell lines after IR [47], but in a following publication, they reported that miR-499a is up-regulated in the LNCaP cell line following IR [48]. Thus, miRNA expression effects may be cell-line dependent, with down-regulation of miR-449a in androgen-dependent prostate cells inducing radioresistance via alterations in cell cycle arrest.

CDK inhibitors can also be targeted by miRNAs, and this interaction is known to be modulated by radiation [82]. Several studies have demonstrated the role of miR-106b on p21 regulation after IR [49, 55] (Fig. 3). Li *et al.* have investigated the expression of miRNAs in LNCaP cells 24 hours after 6 Gy IR, and showed that the expression of several miRNAs was altered. This included miR-106b, which appeared to be down-regulated up to 48 hours following IR. miR-106b is known to target p21, which results in suppression of cell cycle block and promotion of proliferation [49]. miR-106b could therefore be a therapeutic target for the population of radiation-resistant PCa patients who fail to exhibit miR-106b decrease following radiotherapy. Another CDK inhibitor, p27, is targeted by miR-221/222 and miR-24 [83, 84] (Fig. 3). Following irradiation of PCa cells, miR-24 was found to be up-regulated [49], whereas miR-221 and miR-222 were decreased in radiosensitive PC-3 cells. These results were confirmed in PCa tissue using the TCGA dataset [41]. Conversely, miR-221 and miR-222 were found to be increased in radioresistant 22RV1-RR cells [50]. Thus, these miRNAs could play a role in promoting a radioresistant phenotype via negatively regulating CDK inhibitors, and their inhibition may impair the growth of PCa [51]. This axis could show promise for therapeutic interventions.

Finally, radiation response can also be regulated by miRNAs whose targets indirectly affect the cell cycle. In a recent publication, the phenotype of miR-498 was examined in DU145 and LNCaP cells after varying doses of ionizing radiation (1 to 8 Gy) [85]. miR-498 is implicated in proliferation progression of PCa cells and radioresistance by targeting Phosphatase and TENsin homolog (PTEN) (Fig. 3). PTEN is an important cell cycle regulator that suppresses the protein kinase B (AKT) signaling pathway, resulting in inhibition of cell cycle progression. Moreover, down-regulation of miR-498 was shown to reduce PCa cell survival after IR. miR-498 is dysregulated across various cancers, and it is also reportedly up-regulated in PCa cells [86]. Thus, it is suggested miR-498 over-expression could lead to PCa radioresistance through inhibition of PTEN.

### miRNAs and cell death

IR-induced DNA damage causes cell death via multiple mechanisms including mitotic catastrophe, apoptosis, senescence and autophagy [87]. The mode of cell death following IR is determined by a wide variety of factors such as cell type, p53 status, radiation dose or fraction, and tumor oxygenation. In PCa, mitotic catastrophe is considered the dominant mode of cell death and the major determinant of clonogenic cell survival following IR [88]. Mitotic catastrophe is a delayed form of cell death arising from unrepaired DNA damage as a result of dysregulated G2/M cell cycle checkpoint arrest and premature progression into mitosis. Aberrant chromosome separation from centrosome hyper-amplification results in formation of giant polyploid cells, which may go through several rounds of cell division before ultimately undergoing delayed apoptosis. Cell cycle arrest, DNA DSB repair, and cell death are tightly interconnected, and miRNAs may directly or indirectly influence one or all of the above to alter cancer radiosensitivity. As miRNAs affecting cell cycle arrest and DNA repair have been discussed in previous sections, this portion will focus on radiomodulating miRNAs with direct experimental analysis on cell death. One such example is miR-145, which sensitizes both LNCaP and PC-3 PCa cells to IR and predicts patient response to neoadjuvant radiotherapy [54]. Gong *et al*. subsequently determined that decreased clonogenic survival with high miR-145 was not due to changes in apoptosis but rather increased number of cells undergoing mitotic catastrophe as a result of significantly reduced DSB repair. In this sense, Ye *et al.* showed that miR-145 targeted the DNA damage repair associated Helicase-lie transcription factor (HLTF) [89], implicated in radioresistance in cervical cancer [90]. Another target of miR-145, also implicated in radiosensitivity, was SENP1 [91]. SENP1 belongs to the small ubiquitin-like modifier (SUMO)-specific protease family, which deconjugates modified proteins to maintain the level of SUMOylated and un-SUMOylated substrates. In PCa, SENP1 modulates several oncogenic pathways, including AR, c-Jun and Cyclin D1 [92]. Thus, as described in PC-3 cell lines, miR-145 induced cell cycle arrest through the downregulation of SENP1 [91].

miR-32 is a PCa oncomiR which when overexpressed increases radioresistance of PC-3 and DU145 cells, as evaluated using MTT assay, and reduces the percentage of apoptotic cells while inducing autophagy following 2Gy IR [93]. The authors demonstrated miR-32 directly targets DAB2 interacting protein (DAB2IP), the loss of which has previously been shown to promote radioresistance in PCa through enhanced DSB repair, G2/M checkpoint control, and evasion of apoptosis [93, 94]. An opposite effect is observed with radiosensitizer miR-99a in C4-2 cells, with repression of chromatin remodeling protein SNF2H by miR-99a decreasing DSB repair following IR [53]. In addition, overexpression of miR-99a increases PARP (poly ADP ribose polymerase) cleavage, a recognized marker of late apoptosis which is cleaved by caspase-3. The authors suggest that miR-99a decreases DSB repair following IR resulting in increased cell death via apoptosis. Additional miRNAs affecting IR-induced apoptosis are miR-498 and miR-449a, which target PTEN and Rb respectively. miR-449a stimulates radiosensitivity through increased G2/M arrest and higher apoptosis induction, while miR-498 promotes radioresistance through reducing radiation-induced apoptosis indicated by lower caspase3/7 activity [47, 85].

### miRNAs under hypoxia

**Fig. 4:**
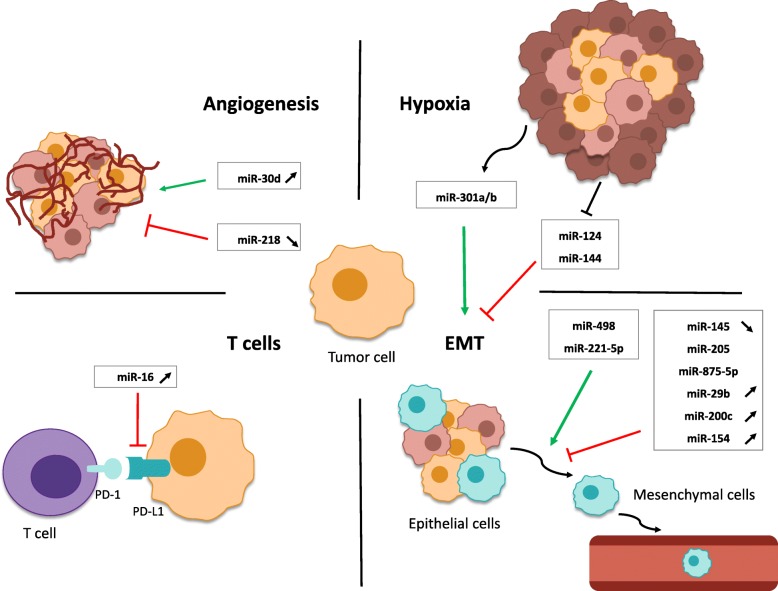
Overview of several actors in the tumor microenvironment modulated by miRNAs involved in irradiation response. Irradiation-modulated miRNAs regulate tumor microenvironment (TME), affecting the radiation response. Angiogenesis, hypoxia, epithelial to mesenchymal transition (EMT) and the immune system are notably affected. Green arrow indicates an induction of the TME actors. Red inhibiting line indicates an inhibition of the TME actors. Up- or down- arrows, next to miRNAs, indicate the expression of miRNAs after irradiation. For miRNAs where no arrow is indicated, means that the miRNA influences the radiation response by modulating TME actors but its up- or down-regulation in PCa cells following irradiation has not been studied. T cells, Lymphocytes T; PD-1, Programmed death 1; PD-L1, Programmed death-ligand 1

Hypoxia is a biological phenomenon associated with tumor progression and acquisition of an aggressive phenotype in solid tumors [95] and radioresistance [96]. The combination of tumor growth and an inadequate tumor vasculature leads to poor perfusion of nutrients and oxygen to the TME and cancer cells [97]. Because of this, tumor cells are exposed to transient hypoxic conditions, which establishes an environment that favors tumor progression and metastasis. Hypoxia is also impacted by IR and modifies radiation response, notably through the alterations in miRNA expression [98]. Numerous studies have suggested that hypoxia triggers EMT in several types of solid tumors, particularly PCa. Hypoxia is known to increase zinc finger protein SNAI1 (SNAIL) and twist family bHLH transcription factor 1 (TWIST1) activity leading to the inhibition of E-cadherin [99]. Wang *et al.* have investigated the relationship of miR-301a and miR-301b to hypoxia and radioresistance. They found that these two miRNAs are hypoxia-responsive and enhance PCa radioresistance by targeting N-myc downstream-regulated gene 2 (NDRG2) [100], a protein which suppresses EMT via the inhibition of signal transducer and activator of transcription 3 (STAT3)/SNAIL signaling [101] (Fig. 4). Hypoxia also induces a decrease of miR-124 and miR-144 [102], which reduces their inhibition of pim-1 oncogene (PIM1) and leads to EMT [103], thereby enhancing radioresistance in an *in vitro* model of PCa [102] (Fig. 4).

Several hypoxia-induced miRNAs have been investigated as they have been thought to regulate radiosensitivity through changes in autophagy and apoptosis. Overexpression of hypoxia-induced miR-301a or miR-301b increased clonogenic survival and lowered radiation-induced apoptosis in LNCaP cells [100]. NDRG2 is a cell-stress response gene targeted by both miR-301a or miR-301b, which regulates numerous survival related molecular pathways including STAT3, SAPK/JNK, Bax, and PI3K/Akt signaling [104]. Conversely, miR-124, miR-144, miR-30a, and miR-205 are all down-regulated in hypoxia, and when overexpressed in PCa cells significantly increased caspase-3 apoptotic activation and inhibited autophagy post IR, while reducing clonogenic survival [42, 102].

### miRNAs in tumor microenvironment

The TME is comprised of a variety of cell types including tumor cells, endothelial cells, immune cells, as well as extra-cellular features such as the extracellular matrix that surrounding cancer cells. All these components that make up the TME components are known to be affected by IR and may therefore modulate therapeutic response [19, 105]. In response to IR, the TME can promote immunogenic cell death through tumor antigen release [106] and induce vascular remodeling to improve radiotherapy efficiency [14], while conversely IR can also induce radioresistance through the promotion of EMT [107]. miRNAs are known to be involved in regulating the TME and radiotherapy response [108, 109].

Tumor growth depends on angiogenesis, a normal physiological process that involves the formation of new blood vessels through proliferation, migration, and differentiation of endothelial cells. Angiogenesis requires coordination of various signaling pathways and cellular activation factors, notably vascular endothelial growth factor (VEGF) [110]. It has been estimated that cancer cells can only reach a maximum volume of 1-2 mm^3^ in the absence of a vascular system [110]. In order for tumors to proliferate and metastasize, angiogenesis must first take place, and miRNAs have been described to regulate this process [111–113]. Lin *et al.* observed that miR-30d promotes angiogenic proliferation and migration, and enhances the ability of PCa cells to recruit endothelial cells via myosin phosphatase targeting subunit 1 (MYPT1)/c-JUN/VEGF-A pathway (Fig. 4). The over-expression of miR-30d is thus associated with advanced cancer progression and an unfavorable prognosis [114]. After IR, miR-30d became decreased in PC-3 cells [41] and it was therefore suggested that miR-30d may increase the efficacy of radiotherapy. In the case of miR-218, its down-regulation contributes to angiogenesis by activating Rapamycin-insensitive companion of mammalian target of rapamycin (RICTOR)/VEGFA pathways [115]. This miRNA has also been shown to be down-regulated after irradiation of a C4-2 cell line [46] (Fig. 4).

Immune cells are critical players within the TME [116]. Immune responses affect cancer development by suppressing or destroying cancerous cells, but these cells eventually develop mechanisms to escape immune surveillance. miRNAs play an important role in the TME by targeting immune cells. For example, miR-16 enhances radiotherapy efficiency in PCa through cytotoxic T cell activation in the TME by suppressing the immune checkpoint regulator PD-L1 programmed death protein 1 (PD-L1) in PCa cells [45] (Fig. 3). miR-16 overexpression in PCa cells after IR may promote radiosensitivity [44]. PD-L1, a ligand expressed on tumor cells, interacts with programmed-death 1 (PD-1) on T cells in order to inhibit CD8+ cytotoxic T lymphocyte survival and proliferation [117]. Thus, PD-L1 down-regulation by miR-16 could enhance immune anti-tumor activity.

### miRNA during the epithelial-mesenchymal transition (EMT)

EMT is characterized by the loss of the polar and adhesive epithelial features, enhanced cell motility and the acquisition of mesenchymal characteristics. It is a complex mechanism which involves modification of various signaling pathways. These pathways are regulated by several transcriptional factors, notably SNAIL, Zinc finger protein SNAI2(SLUG), TWIST 1/2 , ZEB 1/2 [118]. In PCa, miRNAs have been shown to induce EMT by regulating these transcription factors directly [119, 120]. Some miRNAs implicated in EMT inhibition have been found to be down-regulated by IR. Indeed, miR-145 has been described to target ZEB2 [121] and human enhancer of filamentation 1 (HEF1) [122] (Fig. 4), and was shown to have reduced expression after irradiation [46], consequently promoting EMT, bone invasion, and decreasing the effectiveness of radiotherapy. miR-205, essential for the maintenance of the basal membrane epithelium in the prostate gland, is implicated in the modulation of IR response [123, 124]. It has been found to be down-regulated in PCa cells compared to normal prostate tissue [124]. In further studies, the over-expression of miR-145 enhanced radiosensitivity *in vitro* and *in vivo* [68], through targeting ZEB1 which increased E-cadherin expression [125] (Fig. 4). Another miRNA which can radiosensitize is miR-875-5p, which is down-regulated in PCa and enhances tumor control after radiation by preventing EMT, as it indirectly targets ZEB1 [70, 126]. Other miRNAs involved in the inhibition of EMT after IR have been shown to be up-regulated, such as miR-29b and miR-200c [49] (Fig. 4). miR-29b was found to inhibit EMT and subsequently reduce PCa metastasis *in vivo*, by inhibiting the expression of SNAIL [127]. miR-200c, a known tumor suppressor miRNA, also suppresses invasion by directly targeting ZEB1 and ZEB2 [128–130]. Thus, miR-29 and miR-200c might enhance the efficacy of radiotherapy via these mechanisms. Radiation also alters the expression of miR-154 in C4-2 cells [46], and over-expression of this miRNA is known to inhibit EMT by targeting high-mobility group A2 (HMGA2) [131] (Fig. 4).

Radiation response can also be influenced by the expression of miRNAs implicated in the induction of EMT. miR-498 induces a radioresistant phenotype in PCa cells by directly targeting PTEN [85]. A major tumor suppressor, PTEN inhibition promotes cancer metastasis and it is frequently loss during PCa progression, with up to 70 % in late stage [132]. With down-regulation or inhibition of PTEN, AKT can be activated, which increases expression of Vimentin, inhibits expression of E-cadherin by SNAIL [133], and ultimately leads to invasion and migration (Fig. 4). In PCa, proliferation and migration are also enhanced by miR-221-5p which inhibits the tumor suppressor gene suppressor of cytokine signaling protein 1 (*SOCS1*) [134] (Fig. 4). This miRNA was found to be down-regulated in PC-3 cells [41] and up-regulated in 22RV1-RR cells [50] after irradiation. Thus, miR-221-5p might be involved in promoting the radioresistant phenotype observed in these cells. However future mechanistic studies must be performed to definitively determine its role.

### miRNA and Cancer stem cell (CSC)

Accumulating evidences supports that CSC are an important subset of cancer cells that play important roles in radiation response, radioresistance and relapse in PCa [135], but a limited number of experiments described the implication of miRNAs in these processes. CSC populations may be enumerated using cell surface markers such as the adhesion molecule CD44. CD44 expression is associated with radioresistance in PCa cells [136]. Liu *et al.* have identified CD44 and EZH2 as direct target of miR-141 [60]. This miRNA is downregulated in Prostate CSC (PCSC) populations but highly expressed in prostate cancer tissues. In PC-3 and LNCaP cells, expressions of Nanog, Oct4 and EZH2 were correlated with miR-21 under hypoxia [95]. Another study showed that miRNA expression profile in PCSC was dependent on the differentiation stage [137].

### miRNAs in extracellular vesicles

Extracellular vesicles (EVs) are a heterogeneous population of membrane vesicles of various origins, secreted by cells into the extracellular space [138, 139]. The main subtypes of EVs are microvesicles and exosomes, which are distinguished based upon their biogenesis, release pathways, size, content, and function. Exosomes are small vesicles (50 – 150 nm in diameter) secreted by almost all cell types, tumoral or not. They are formed via inward budding of endosomal membrane during maturation of multi-vesicular endosomes (MVEs) and later secreted when the MVEs fuse with the plasma membrane [138]. These small vesicles play a role in intercellular communication by transferring their contents in order to influence many physiological and pathological functions in the recipient cell [140, 141]. It has been suggested that EVs may be involved in cancer progression and recurrence in this way [142]. In human body fluids, such as blood and urine, miRNAs are protected from endogenous RNAse activities by their interaction with proteins or lipids, or their encapsulation into exosomes [140, 143, 144]. PCa-derived exosomes have been found to be released in diverse biofluids and could serve as a liquid biopsy for PCa [145], which will be discussed in more detail in the following section. Cellular stress, which can be caused by IR, can affect the molecular composition of miRNAs in exosomes, and also increase loco-regional exosome secretion.

In a recent study, exosomal miRNAs in the sera from 11 PCa patients (6 intermediate-risk and 5 high-risk disease) treated with radiotherapy were analyzed [146] (Table 2). A comparative analysis was performed looking at the expression of miRNAs before and after radiotherapy. Using Nanosight, they showed that exosome concentrations in serum were higher in PCa patients compared to healthy individuals, and this trend held true following IR. After radiotherapy, miR-21-5p and let-7a-5p were found to be significantly higher in serum-derived exosomes of high-risk (*i.e.* Gleason 7 or higher (3+4, 4+4, 4+5 or 8) and pathological T stage (cT1c, cT3a, pT3 or pT3a)) PCa patients compared to intermediate-risk (*i.e.* Gleason 7 or under (3+3, 3+4 or 4+3) and pathological T stage (cT2a, cT2b or cT1c)) PCa patients. However, due to the small sample size, these results require validation within larger patient cohorts.

**Table 2:** exosomal miRNAs in radiotherapy response in prostate cancer

miRNA	Biomarkers	Biofluids	Main results	Cohorts	References	Targets
let-7a-5p	Efficacy of RT	Serum exosomes	Upregulated in exosomes of high-risk PCa patients after RT compared to intermediate-risk PCa patients	11 PCa patients (6 intermediate-risks, 5 high-risk disease)	[146]	-
miR-21	Diagnostic/ efficacy of RT					PTEN [147], MARCKS [148], ANP32A, SMARCA4 [149]
miR-200c-3p	Efficacy of CIRT	Serum exosomes	Upregulation in exosomes is associated with a good response to CIRT	8 PCa patients (3 intermediate risk, 2 high risk, 3 very high risk)	[150]	ZEB1, ZEB2 [128]
miR-323-3p						p73 [151], AdipoR1 [152]
miR-379-5p						-
miR-409-3p						PHC3, RSU1,TUSC1 [153]
miR-411-5p						-
miR-493-5p						c-Met, CREB1, EGFR [154]
miR-494-3p						CXCR4 [155]
miR-543						RKIP [156]
miR-654-3p						AR [74]

*RT* radiotherapy, *CIRT* carbon ion radiotherapy, *PCa* prostate cancer, *PTEN* phosphatase and TENsin homolog, *MARCKS* myristoylated alanine-rich protein kinase c substrate, *ANP32A* acid nuclear phosphoprotein 32 family member A, *SMARCA4* SWI/SNF related matrix associated actin dependent regulator of chromatin subfamily A member 4, *AdipoR1* Adiponectin receptor 1, *PHC3* polyhomeotic homolog 3, *RSU1* Ras suppressor protein 1, *TUSC1* tumor suppressor candidate 1, *CREB1* CAMP responsive element binding protein 1, *EGFR* epidermal growth factor, *CXCR4* CXC chemokine receptor 4, *RKIP* Raf kinase inhibitor protein, *AR* androgen receptor

Yu and colleagues have also evaluated exosomal miRNAs to monitor the efficacy of carbon ion radiotherapy (CIRT) [150] (Table 2), which has superior dose distribution, higher linear energy transfer and increased biological effectiveness compared to conventional photon-based external beam radiation. To do so, the authors analyzed exosomal miRNAs from the sera of 8 patients with localized prostate cancer exposed to CIRT. They observed deregulated expression of 57 miRNAs after CIRT, and 25 which correlated with PSA (prostate specific antigen). They next showed that patients responding to CIRT, based on PSA < 0.2 ng/mL after CIRT, had higher expression of 9 miRNAs (miR-200c-3p, miR-323a-3p, miR-379-5p, miR-409-3p, miR-411-5p, miR-493-5p, miR-494-3p, miR-543 and miR-654-3p) in their sera compared to non-responders (PSA > 0.2 ng/mL after CIRT). They also performed a comparison of miRNA content in exosomes before and after CIRT, which correlated miR-379-5p and miR-654-3p expression with an effective response to CIRT. This study is the first to explore the modulation of exosomal miRNAs expression in PCa after CIRT exposure. However, these results should be interpreted with caution due to the small cohort of patients and may be specific to CIRT treatment.

It has been shown that IR treated cells secrete exosomes that can be taken up by non-irradiated neighboring cells and induce biological changes via the bystander effect [157, 158]. These IR-induced exosomes may promote radioresistance, leading to tumor progression and the formation of a pre-metastatic niche. Previous examples have indeed shown a resistance transfer by exosomes in breast cancer carcinoma [159, 160], head and neck cancer [161] and glioblastoma [162]. The presence of miRNAs in the circulation may make them a promising source of biomarkers for improving PCa management and monitoring treatment response following radiotherapy.

### miRNAs as biomarkers

Improved clinical outcomes for patients with PCa are highly dependent on the tests we use to detect and monitor disease. Indeed, physicians and scientists are limited by the current technologies available at hand to understand this disease and its trajectory. In PCa, the discovery of PSA and integration of magnetic resonance imaging, have made huge strides in assisting with the diagnosis of PCa. Despite this, histopathological analysis of a biopsy specimen remains the gold standard test for PCa diagnosis and it is required for prognostication and treatment planning [163]. However, the core needle biopsy is not without significant side effects. From pain and bleeding, to infection with risk of progression to sepsis [164], these complications are not to be taken lightly. As such, prophylactic antibiotics are routinely administered to prevent such risks, but in 2019 with a rise of antibiotic-resistant bacterial strains, this practice must also not be overlooked. There is a significant clinical need to move toward developing non-invasive tests for cancer diagnosis, prognosis and prediction.

The field of circulating biomarkers (*i.e.* from whole blood, serum, plasma, and urine) has exploded in recent years. These “liquid biopsies” are collected from simple, non-invasive blood draws and urine samples, which harbor minimal (if any) side effects for patients. Once fine-tuned, the possibilities are really endless for which clinical setting circulating biomarkers could be used in – diagnostic, predictive, prognostic – and they would open doors to new ways of monitoring the progression of disease and response to therapies.

In PCa, there are a few commercially-available circulating biomarker tests. ExoDx Prostate (IntelliScore) [165] and PCA3 [166, 167] utilize urine samples, collected after a digital rectal exam (DRE). They use a gene-based or non-coding RNA signature, respectively, to help clinicians identify patients who are more likely to harbor clinically-significant PCa (i.e., Gleason score 7 or higher), and thus would benefit from a prostate biopsy. The 4Kscore Test [168, 169] is blood-based test that uses a panel of four kallikreins to predict the likelihood of a clinically-significant cancer. Although there are no commercially-available miRNA signatures, there are many that have been described in the literature, as previously reviewed [170, 171]. A few studies of note will be described in detail here. Alhasan *et al.* found a 5-miR (miR-200c, miR-605, miR-135a*, miR-433, and miR-106a) serum signature to identify patients with very high-risk PCa (*i.e*. Gleason 8 (4+4 or 5+3) and Gleason 9) [172]. Hoey *et al.* identified a 4-miR (miR-20a, miR-20b, miR-106a, and miR-106b) signature that stratifies PCa patients into low- and high-risk categories after radical prostatectomy [173]. Post-radical prostatectomy is a particularly important clinical setting for circulating biomarkers to detect biochemical recurrence and identify patients who would benefit from adjuvant therapy. A serum-based 3-miR (miR-223, miR-24, and miR-375) score was developed by Liu *et al*. to identify males on active surveillance who have indolent PCa compared to those with aggressive disease who need to be treated [174]. miR-1246 has been described as an serum exosomal biomarker to distinguish benign and aggressive PCa [175]. miR-1290 and miR-375 in the plasma were associated with significantly poorer overall survival in patients with castrate-resistant metastatic PCa [176]. Jeon *et al.* found a urine-based 7-miR (miR-3195, let-7b-5p, miR-144-3p, miR-451a, miR-148a3p, miR-512-5p, and miR-431-5p) signature that identifies high-risk PCa patients with high accuracy (AUC of 0.74, 95% CI = 0.55-0.92) [177], and this miRNA signature was stable over time. It is of particular relevance to note the unique superiority of a urine-based biomarker for PCa, as Pellegrini *et al*. discovered that urine collected after a DRE is enriched in prostate-specific markers [178]. They found that post-DRE urine EVs contain prostate-derived RNAs, which were able to distinguish patients with low-risk (Gleason 6) and intermediate- and high-risk (Gleason 7 and above) PCa.

In regards to biomarkers of radiotherapy response, Zedan *et al.* identified miR-221 to be decreased in patient plasma after RT [179]. As previously mentioned, miR-221 expression is decreased in radiosensitive PC-3 cells [49] and increased in radioresistant 22RV1-RR cells [50]. Thus, the modulation of this miRNA might be investigated as a biomarker to monitor radiotherapy response. Further studies need to be performed to confirm that miR-221 could be used since biomarkers from biofluids do not necessarily reflect the miRNA profile of the tumor cells.

The temporal stability of miRNA in biofluids is a significant feature of an ideal biomarker. Since miRNA biomarker signatures can remain stable over time, an important application is their potential to non-invasively monitor disease progression or treatment response (*i.e*. before and after surgery, radiotherapy, hormone therapy and chemotherapy, as well as throughout the course of treatment). Various circulating miRNAs have been described for their predictive biomarker utility to distinguish patients who would benefit from treatment, thus personalizing management decisions [180–184]. Circulating miRNAs have even been used in the setting of identifying which patients have a higher risk of treatment toxicity. A study by Higgins *et al.* found that lower expression of miR-6821 and miR-1290 in the serum of patients with squamous cell carcinoma of the head and neck was associated with increased probability of developing acute and late radiation-induced toxicity [185]. A study by Lin *et al.* looking at circulating miRNAs in CRPC patients treated with docetaxel, found that miRNAs isolated from patient blood were associated with PSA response and overall survival using pre-docetaxel levels, or the direction of post-docetaxel change from baseline levels [181]. However, to the best of our knowledge, there are no circulating miRNA biomarkers described for the purpose of predicting response to or benefit of receiving radiotherapy in prostate cancer.

There are various characteristics of miRNAs that make them particularly attractive candidates for biomarker development, as previously reviewed by Schwarzenbach *et al.* [171]. miRNAs are highly stable in the blood and urine due to their association with proteins (such as AGO) or their encapsulation in EVs [171, 186]. After blood draw, miRNAs can remain stable after incubation at room temperature and after undergoing repeat freeze-thaw cycles [186]. They can easily be detected with a standard qRT-PCR, which offers high sensitivity and specificity for a relatively low cost compared to other biomarker assays [171]. NGS has the ability to identify novel miRNA, and distinguish miRNA with similar sequences and those of splice variants (isomiRs) [171]. NGS deep sequencing can also assess genome-wide expression, however, this improved technology comes with a high financial cost [171]. Last but not least, miRNAs can be readily found in all body fluids, not just blood and urine [187].

Before non-invasive miRNA biomarkers can safely and effectively be translated into clinical studies and practice, various logistical issues must be addressed. For one, a standardized test for assessing the expression levels of blood and/or urine miRNA must be established. There is known to be large variability between platforms [188], even though individual platforms show very high correlations between technical replicates [189]. Aside from downstream technologies, laboratory reagents and protocols must also be standardized. It has been found that the specific anticoagulants (*i.e.* EDTA, heparin, citrate) used in blood collection tubes can affect downstream qRT-PCR analysis [190]. Indeed, determining which miRNA signature from the literature is superior to be used for the desired clinical setting, and reproducibility of this particular miRNA signature must be determined using large, prospective, multi-institutional cohorts.

Although there is still much work to be done before non-invasive miRNA biomarkers can begin to be used in the clinical setting, miRNAs show significant promise as a future non-invasive biomarker for cancer diagnosis, treatment prediction, and prognostication.

### Future perspectives

There are currently very limited publications on the role of miRNAs in regulating PCa radiotherapy response and mediating PCa radioresistance [40, 41, 43, 44, 46, 49, 50]. Moreover, the majority of these studies are performed on cell lines *in vitro*, and miRNAs found altered by IR in one study are not necessarily observed in others. This could be due to differences in IR doses and delivery methods, or variations between cell lines. Future studies are needed to explore miRNAs dysregulated by IR using not only PCa cells lines but also validation with patient samples.

In addition, the functional role of these miRNAs in cellular response to IR is not well understood and is largely in the infancy of discovery. Although miRNA can target a multitude of mRNAs, their function may be due to their regulation of only a select few. Thus, potential targets of miRNAs involved in radioresistance need to be thoroughly investigated in order to delineate true contributors to resistance so that novel therapeutic interventions will be focused on downstream components to overcome radioresistance. To our knowledge, there are no reports on miRNAs involved in ROS signaling, and only a few on hypoxia signaling and angiogenesis, in PCa following radiotherapy. Furthermore, no investigations have focused on the effect of exosomal miRNAs from PCa modulated by IR, and their impact on the tumor microenvironment. Thus, it will be beneficial for investigations to fill-in these missing gaps in radiobiology, for example the role of IR-induced exosomal miRNAs on immune escape. Indeed, Vignard *et al.* recently report that several miRNAs from melanoma-derived exosomes participate to tumor immune escape by reducing CD8+ T cell response [191]. This might also be the case in prostate cancer following alteration of exosomal miRNA expression, which could lead to an enhancement in radioresistance.

Finally, it is essential to identify biomarkers to support the use of PSA in diagnosis and disease monitoring to improve accuracy. The detection of changes in circulating miRNAs shows promise as a prognostic indicator to differentiate between indolent and aggressive disease, and to predict radiation response in PCa to individualize treatment for patients.

## Conclusion

Radiation therapy is a critical modality of treatment for prostate cancer patients. Despite its delivery with curative intent, radioresistance frequently occurs and the underlying mechanisms are poorly understood. It is well characterized however, that ionizing radiation induces aberrant expression of miRNAs. These miRNAs, which are important regulators of gene abundance, play a role in radioresistance by modulating key cellular pathways that mediate radiation response. Overcoming radioresistance is a significant clinical challenge in prostate cancer management. Thus, identifying non-invasive biomarkers to inform treatment decisions in the clinic are desperately needed, and it is likely that miRNAs could be useful for this purpose. Furthermore, highlighting the targets of deregulated miRNAs will open doors for future therapies to sensitize prostate tumors to radiotherapy and improve tumor control. Various technologies may prove useful in this setting: miRNA mimics to overexpress tumor suppressor miRNAs decreased by radiotherapy, or small interfering RNAs and antisense oligonucleotides to inhibit oncomiRs up-regulated after radiotherapy. However, the delivery of these future treatments is still challenging due to the plethora of downstream targets that each miRNA can regulate. Therefore, it is imperative we untangle the cellular complexities in PCa radiotherapy resistance in order to improve PCa treatment and tumor control. Understanding the role of miRNAs in this setting brings us one step closer to achieving this goal and ultimately improving patient outcomes.

## Data Availability

Not applicable.

## Abbreviations

3’UTR: 3’ untranslated region
Ack1: Activated CDC42 kinase 1
ADT: Androgen Deprivation Therapy
AGO: Argonaute
AKT: Protein kinase B
AR: Androgen receptor
AREs: Androgen Response Elements
ATM: Ataxia-Telangiectasia Mutated
BRCA1: Breast cancer susceptibility gene 1
Cdc25A: Cell division cycle 25A
CDKs: Cyclin dependent kinases
CHK1: Checkpoint kinase 1
CIRT: Carbon ion radiotherapy
CREPT: Cell cycle-related and expression-elevated protein in tumor
CRPC: Castration-Resistant Prostate Cancer
CSA: Cockayne syndrome protein A
CSC: Cancer Stem Cell
DAB2IP: DAB2 interacting protein
DCs: Dendritic cells
DDR: DNA damage response
DNA-PK: DNA-dependent protein kinase
DRE: Digital Rectal Exam
DSBs: Double-strand breaks
EGFR: Epidermal growth factor receptor
EMT: Epithelial to mesenchymal transition
EVs: Extracellular vesicles
EZH2: Enhancer of zeste 2 polycomb repressive complex 2 subunit
HEF1: Human enhancer of filamentin 1
HIF-1α: Hypoxia-inducible factor 1α
HLTF: Helicase-lie transcription factor
HMGA2: High-motility group A2
HR: Homologous recombination
IR: Ionizing radiation
MAD2L2: Mitotic arrest deficient 2 like 2
MCL1: Myeloid cell leukemia 1
miRNAs: microRNAs
MVEs: Multi-vesicular endosomes
MYPT1: Myosin phosphatase targeting subunit 1
ncRNAs: Non-coding RNAs
NDRG2: N-myc downstream-regulated gene 2
NGS: Next generation sequencing
NHEJ: Non-homologous end joining
PARP: Poly ADP ribose polymerase
PCa: Prostate cancer
PCSC: Prostate Cancer Stem Cell
PD-1: Programmed-death 1
PD-L1: Programmed-death ligand 1
PIM1: Pim-1 oncogene
PKCε: Protein kinase C epsilon
PSA: Prostate specific antigen
PTEN: Phosphatase and TENsin homolog
RAD23B: RAD23 homolog B
Rb: Retinoblastoma protein
RICTOR: Rapamycin-insensitive companion of mammalian target of rapamycin
RISC: RNA-induced silencing complex
ROS: Reactive oxygen species
RR: Radioresistant
SGPP1: Sphingosine-1-phosphate phosphatase 1
SLUG: Zinc finger protein SNAI2
SNAIL 1: Zinc finger protein SNAI1
SNF2H: SWI/SNF-related matrix-associated actin-dependent regulator of chromatin subfamily A member 5
SOCS1: Suppressor of cytokine signaling protein 1
STAT3: Signal transducer and activator of transcription 3
SUMO: Small Ubiquitin-like Modifier
TME: Tumor microenvironment
Twist 1/2: Twist family bHLH transcription factor 1/2
VEGF: Vascular endothelial growth factor
XLF: XRCC4-like factor
XPC: Xeroderma pigmentosum complementation group C
ZEB1/2: Zinc finger E-box binding homeobox 1/2
